# Preliminary Findings Show Maternal Hypothyroidism May Contribute to Abnormal Cortical Morphology in Offspring

**DOI:** 10.3389/fendo.2016.00016

**Published:** 2016-02-25

**Authors:** Julieta E. Lischinsky, Jovanka Skocic, Hayyah Clairman, Joanne Rovet

**Affiliations:** ^1^Institute for Biomedical Sciences, The George Washington University, Washington, DC, USA; ^2^Center for Neuroscience Research, Children’s National Medical Center, Washington, DC, USA; ^3^Neuroscience and Mental Health Program, The Hospital for Sick Children (SickKids), Toronto, ON, Canada; ^4^Department of Pediatrics, University of Toronto, Toronto, ON, Canada; ^5^Department of Psychology, University of Toronto, Toronto, ON, Canada

**Keywords:** hypothyroidism, pregnancy, thyroid hormone, cortex, MRI, FreeSurfer

## Abstract

In rodents, insufficient thyroid hormone (TH) gestationally has adverse effects on cerebral cortex development. Comparable studies of humans examining how TH insufficiency affects cortical morphology are limited to children with congenital hypothyroidism or offspring of hypothyroxinemic women; effects on cortex of children born to women with clinically diagnosed hypothyroidism are not known. We studied archived MRI scans from 22 children aged 10–12 years born to women treated for preexisting or *de novo* hypothyroidism in pregnancy (HYPO) and 24 similar age and sex controls from euthyroid women. FreeSurfer Image Analysis Suite software was used to measure cortical thickness (CT) and a vertex-based approach served to compare HYPO versus control groups and Severe versus Mild HYPO subgroups as well as to perform regression analyses examining effects of trimester-specific maternal TSH on CT. Results showed that relative to controls, HYPO had multiple regions of both cortical thinning and thickening, which differed for left and right hemispheres. In HYPO, thinning was confined to medial and mid-lateral regions of each hemisphere and thickening to superior regions (primarily frontal) of the left hemisphere and inferior regions (particularly occipital and temporal) of the right. The Severe HYPO subgroup showed more thinning than Mild in frontal and temporal regions and more thickening in bilateral posterior and frontal regions. Maternal TSH values predicted degree of thinning and thickening within multiple brain regions, with the pattern and direction of correlations differing by trimester. Notably, some correlations remained when cases born to women with severe hypothyroidism were removed from the analyses, suggesting that mild variations of maternal TH may permanently affect offspring cortex. We conclude that maternal hypothyroidism during pregnancy has long-lasting manifestations on the cortical morphology of their offspring with specific effects reflecting both severity and timing of maternal TH insufficiency.

## Introduction

Thyroid hormone (TH) is essential for normal brain development from the start of pregnancy until the first few years of life (1, 2). Because the fetal thyroid system matures late in gestation, maternal TH is needed at the beginning of pregnancy for normal fetal brain development and serves as a supplementary, albeit essential, source later (3). However, if the mother’s TH supply is inadequate due to clinical or subclinical hypothyroidism, offspring neurodevelopment will be compromised leading to a reduced IQ, cognitive and learning deficits, and behavior problems (4–10). Although these consequences are mostly associated with early gestational TH deficiencies (11), adverse effects of later TH insufficiency are also known (12). To date, most of the research has been based on global endpoints (e.g., IQ), which may be insensitive to subtle manifestations or localized brain changes (3, 13), while little direct evidence exists on offspring brain development (14, 15).

In rodents, abundant research shows a wide range of brain abnormalities in the progeny when pregnant dams are rendered TH-insufficient at different stages of pregnancy (11, 16, 17). Diverse cortical abnormalities (18, 19) reflecting both severity and timing of maternal TH deficiency (20) include neuronal migration defects, atypical layering (21, 22), reduced growth of axons and dendrites (23, 24), and increased apoptosis (25). These effects are generally localized to middle and posterior brain regions, especially parietal and occipital cortices (26–29), which underlie sensorimotor, visual, and auditory functions (2, 30). By contrast, the frontal lobes, which are important for executive processing and attention require TH in the postnatal period (31), signifying a posterior-to-anterior progression of TH need within the cortex.

Recent advances in the analysis of MRI scans to evaluate cortical morphology now make it possible to directly study the human cortex. The available automated programs serve to recreate and measure surface features of the cortex, such as cortical thickness (CT) and surface area (SA) (32), which differ as to underlying genetic determinants, cellular processes, and timing effects (33). CT, the most commonly studied parameter, refers to the distance between the brain’s inner white-matter (WM) and outer gray-matter (GM) (i.e., pial) surfaces measured across the entire cortex (34). A number of neurodevelopmental conditions, such as autism spectrum disorder, attention-deficit hyperactive disorder, human X monosomy, fetal alcohol spectrum disorder, and William’s syndrome show a wide variety of CT abnormalities (35–42), which are typically attributed to defects in neuronal migration or cortical layering (43, 44). Recently, we reported that children with congenital hypothyroidism (CH), who are TH-deficient in the period from late gestation until the first month or two of life, show cortical thinning and thickening in diverse regions with specific effects reflecting initial disease severity and predicting current neuropsychological functioning (45). By contrast, studies from the Netherlands examining children’s cortical morphology report normal CT and SA measurements if mothers had hypothyroxinemia in early pregnancy (i.e., low thyroxine or T4 but normal thyroid-stimulating hormone or TSH) (46) but cortical volumes were reduced if mothers’ free T4 levels were at the low or high extremes of the normal range (47). Not known, however, is whether children exposed to insufficient TH during gestation due to inadequately treated clinical hypothyroidism in the mother show abnormal cortical development.

The current study examined CT in young adolescents from a cohort born between 1996 and 2001 and regularly followed since infancy. Included were offspring of women treated for clinical hypothyroidism in pregnancy (HYPO) and of euthyroid women (controls). At time of entry into our project, public knowledge that HYPO required a substantial increase in dosage of TH was not available (48) nor were guidelines for managing maternal HYPO (49, 50). Consequently, most mothers were undertreated while their children were exposed to some gestational TH insufficiency. We presently performed CT studies on archived structural scans in order (i) to determine if HYPO and controls differ and identify where differences reside and (ii) to examine if CT and maternal hypothyroidism severity are related. We hypothesized that relative to controls, HYPO would show atypical cortical morphology that would reflect severity and timing of maternal hypothyroidism. We did not have specific predictions as to where regionally or in what hemisphere differences would occur and their extent; however, we expected effects to be relatively posterior given rodent findings showing a posterior-to-anterior progression of TH need in the brain.

## Materials and Methods

### Participants

The current sample consisted of 48 healthy children (30 males, 18 females) whose mothers were recruited between 1996 and 2001 during pregnancy or shortly after delivery. Children were previously studied at 6, 12, and 18 months and 5 years of age. Recently at 10–12 years, they participated in a large-scale study investigating their neuropsychological characteristics (particularly memory) and brain anatomy based on structural and functional MRI scans (14, 15).

The HYPO group included 24 from a birth cohort consisting of 66 children whose mothers were originally recruited during pregnancy via posters placed in local endocrinologists and obstetricians’ offices, while a small subset was also directly recruited from the records of an antenatal counseling service at The Hospital for Sick Children (SickKids) for teratogen and medication exposure. Most non-participants were lost to follow-up or not able to participate due to time constraints. Mothers of current and non-participants did not differ in TH levels or socioeconomic status (SES). Their children did not differ in gestational age or birth weight.

The 24 HYPO cases were born to 23 women; one mother, who was diagnosed in the first pregnancy, contributed two children. Most women (83%) were treated with thyroxine prior to pregnancy, while 17% began it during pregnancy. The majority was diagnosed with Hashimotos thyroiditis, while two women had thyroid ablation, one for hyperthyroidism and one for thyroid cancer. Thyroid function test results (primarily TSH) were available from the pregnancy records of all but one woman, who however was told by her physician that her TSH levels were extremely elevated. As some women only began treatment during the second trimester, their second-trimester values were carried back to replace their missing data. Based on current trimester-specific ranges for TSH (51), two women had normal TSH levels during each trimester, whereas the remainder had an elevated value in one or more trimesters. The HYPO group was also stratified into Mild and Severe subgroups based on a median split of mothers’ TSH levels from the first and/or second trimesters (TSH + 8.8 levels). For 15 children, newborn TSH values obtained from provincial screening-lab records were all in the normal range (mean = 4.11 mU/l, range = 1.5–12.6 mU/l).

Controls were 24 typically developing youth (15 males, 9 females) from the same memory study and individually matched with HYPO for sex, age (+6 months), and SES (same class value except for one pair differing by one class level). Twenty-one were from the original birth cohort, one was a participant in a previous study in the lab, and two were newly recruited for the memory study. All mothers reported not being hypothyroid in pregnancy or since delivery. At the time when the birth cohort was originally recruited, thyroid function testing was not routinely performed in pregnancy in our province; however, a small subset was tested by one of the participating obstetricians and all had normal-range TH results. Newborn TSH values available on 13 control children were all in the normal range (mean = 3.68 mU/l, range = 1.2–11.0 mU/l).

### Procedures

As part of the larger study, all children received over the course of 2 days, an extensive battery of neuropsychological tests and questionnaires and structural and functional MRI scans, approximately 2 weeks apart (14). Tests included the two-subtest version of the Wechsler Abbreviated Scale of Intelligence (WASI) (52), which yielded a Full Scale IQ (FSIQ) score from these two subtests, Vocabulary and Matrix Reasoning subtests, that were given. Results from remaining tests are reported elsewhere (14, 15). Parents provided informed consent while children gave assent. All procedures were approved by the Research Ethics Board at SickKids.

### Imaging Acquisition and Processing

Whole-brain T1 images were acquired in a 1.5-T GE Signa Excite research scanner with an anatomical Inversion Recovery Prepared T1-weighted fast spoiled gradient echo sequence. The following acquisition parameters were used: repetition time = 10.09 ms, echo time = 4.2 ms, inversion time = 400 ms, flip angle = 20°, acquisition matrix = 256 mm × 192 mm, voxel size = 0.9375 mm × 0.9375 mm, and slice thickness = 1.5 mm. Acquisition time was 7 min. All scans were examined by a staff neuroradiologist who reported on incidental brain abnormalities. Any child with a scan indicating a significant neuroradiological abnormality that would affect cortical measurements was excluded. As one child with a large right frontal dysembryoplastic neuroepithelial tumor was removed, 23 HYPO and 24 control scans were available for cortical morphology analysis.

Image analysis was performed on a Linux Fedora operating system using FreeSurfer Image Analysis Suite version 4.4 (53). When this study originally began, this version was the most current and used in other research in our lab (45). FreeSurfer consists of a series of automated algorithms that reproduce the brain’s inner and outer surfaces and measure distances between these surfaces on a point-to-point basis for the entire cortical mantle. Processing steps included the following: transforming each subject’s native brain into Talairach space, intensity normalization, removal of non-brain tissue, and segmentation of GM/WM tissue (54), and smoothing using a Gaussian kernel with a full width half maximum of 10 mm. Total processing time per subject was 24 h. Initially, each cortex was manually inspected for quality control and motion by Jovanka Skocic, who was blinded to participant group status. Any scan showing extensive motion was eliminated from the analysis. We also checked surface extraction for systematic errors. In the HYPO group, one scan was removed for excessive motion yielding a final sample size of 22 HYPO and 24 controls.

Whole-brain vertex-based CT statistical analyses were performed using FreeSurfer’s Qdec program. These were conducted separately by hemisphere. Also provided by FreeSurfer were measures of surface area and gyrification and intracranial, GM, WM, and cerebrospinal fluid volumes. For current purposes, only CT and total intracranial volume data were used. For all analyses, the Destrieux Atlas identified locations of significant clusters.

### Data Analyses

Demographic and volumetric data were originally analyzed in SPSSv21 using *t*- and chi-square tests. Within FreeSurfer, two sets of between-group comparisons were performed using the General Linear Model analysis program, one contrasting HYPO and control groups and the other, Mild and Severe HYPO subgroups. To examine relations between trimester-specific maternal TSH levels and CT, regression analyses were performed on the scans of children whose mothers had TH data. All analyses were conducted across the entire cortical surface of ~80,000 vertices per hemisphere. Since we had no prior hypotheses on specificity, we did not examine within specific regions or lobes. The threshold for group comparisons was set at *p* < 0.05 and for regressions, at *p* < 0.01. To correct for multiple comparisons, we used a cluster-wise procedure involving the Monte Carlo null-z simulation with 5000 permutations. However, we only report group-comparison results at *p* < 0.001 to further reduce the possibility of Type 1 errors. For all regions showing regression effects, we also extracted individual CT values at the peak vertex of each significant cluster and in SPSSv23, correlated the derived CT values with trimester-specific maternal TSH levels.

## Results

### Demographic and Total Brain Volume Data

Table 1 providing the mean demographic data for HYPO (*n* = 22) and control (*n* = 24) groups shows that they did not differ in age, sex, SES, or handedness. All participants scored above 80 in IQ, although HYPO’s scores were on average 3.7 points below control values, but the difference was not significant. For the entire sample, total intracranial volumes ranged from 1351.1 to 2085.5 cm^3^ and did not differ between groups. Because groups did not differ on any of the above indices, we did not include any covariates in the subsequent analyses.

**Table 1:** **Demographic data for HYPO and control groups**.

	HYPO^a^ (*n* = 22)	Control (*n* = 24)	*p*-value
Age (years)	10.3 (0.6)	10.5 (0.71)	0.28
Sex (male/female)	14/8	15/9	
Socioeconomic status^b^	1.86 (0.77)	1.83 (0.86)	0.9
Handedness (right/left)^c^	21/1	23/1	
Full Scale IQ	113.1 (12.6)	116.8 (10.2)	0.27
Intracranial volume	1774.5 (180.5)	1750.0 (152.5)	0.4
TSH (mU/l)^d^			
Trimester 1	8.70 (6.71)		
Trimester 2	6.30 (8.50)		
Trimester 3	3.24 (4.17)		
Free T4 (nmol/L)^e^			
Trimester 1	11.05 (2.65)		
Trimester 2	13.02 (2.17)		
Trimester 3	12.54 (2.15)		
Thyroxine dosage (μg/day)	103.5 (41.8)		
Range	50–200		

*^a^This number represents the remaining sample after the two cases with unacceptable scans were eliminated*.

*^b^Based on Hollingshead and Redlich’s (1975) 5-point rating scale for socioeconomic status (SES) derived from occupations and educational levels of both parents; this information was ascertained from a case-history questionnaire given to parents. A rating value of 1 signifies the highest SES level and of 5, the lowest*.

*^c^Based on a 10-item questionnaire completed during by children during the neuropsychological assessment*.

*^d^Based on 21 women for trimester 1, 19 for trimester 2, and 16 for trimester 3; values elevated in all trimesters*.

*^e^Based on 14 women for trimester 1, 12 for trimester 2, and 10 for trimester 3; values low but not significantly so for each trimester*.

Table 1 also provides for the HYPO group, mean TSH and T4 level values from each trimester as well as the initial dose level of thyroxine in pregnancy. TSH values were consistently elevated, whereas free T4 levels were normal or below but not significantly low. Even though dose levels varied among the women, their dosages did not directly reflect TSH values. For example, the woman with the most aberrant TSH values was receiving 125 μg/day l-thyroxine, which was relatively high.

### Between-Group Comparisons of Cortical Thickness

The HYPO group included only children born to women with elevated TSH levels in pregnancy since the two women with normal TSH levels in pregnancy were technically not gestationally hypothyroid (likely because their treatment was sufficient to maintain their TSH levels in the normal range). The child whose mother was told by her physician that her levels were extremely elevated was included. The final sample size for these analyses was 20 HYPO and 24 controls.

**Figure 1:**
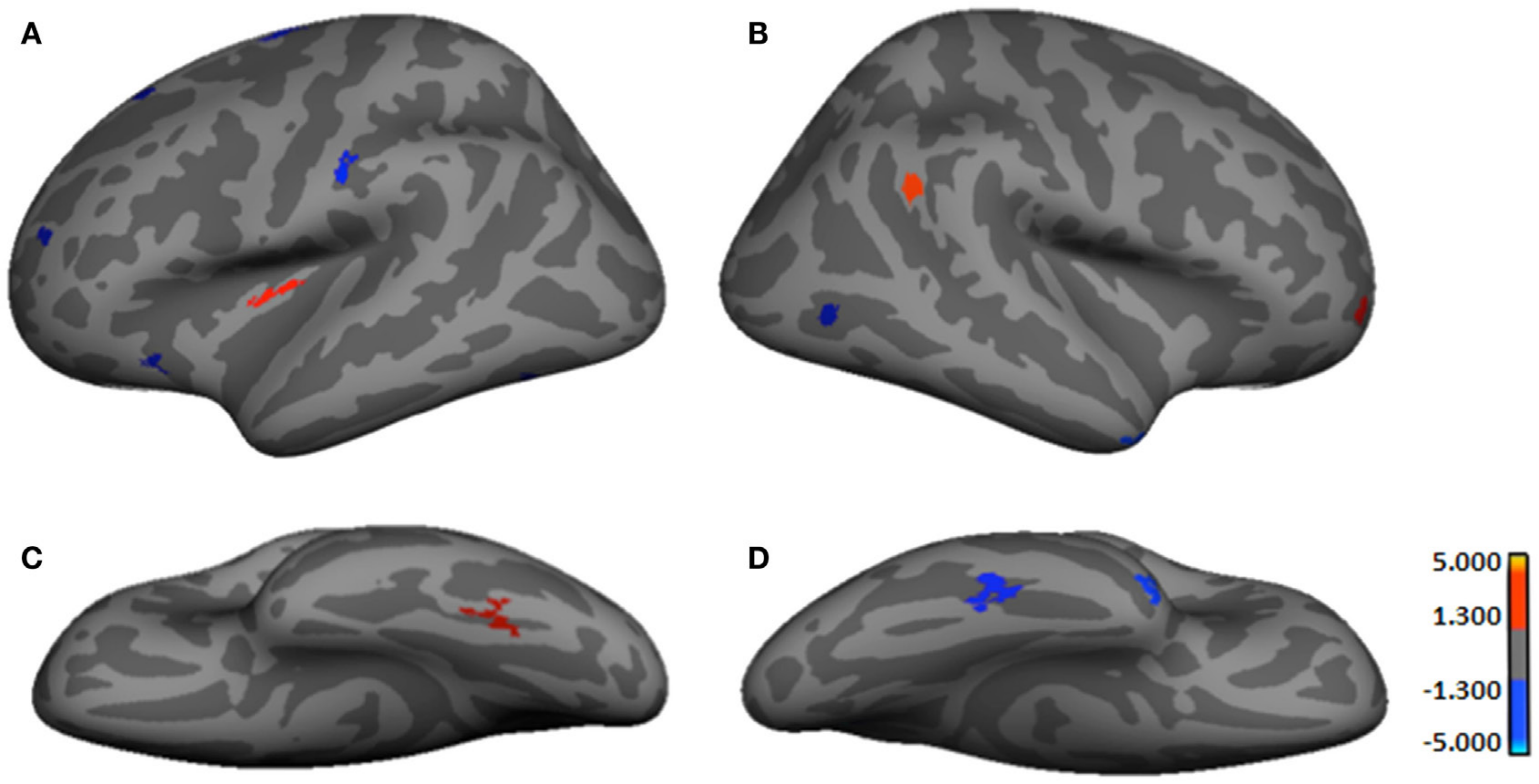
**Regions showing significant cortical thickness differences between groups**. **(A)** Left lateral-view spatial map showing regions of significant thinning (red) and thickening (blue) in HYPO. **(B)** Right lateral-view spatial map showing regions of significant thinning (red) and thickening (blue) in HYPO. **(C)** Left inferior-view spatial map showing regions of significant thinning (red) in HYPO. **(D)** Right inferior-view spatial map showing regions of significant thickening (blue) in HYPO. As per heat bars, regions depicted in red signify thinning in HYPO (Control > HYPO) and regions shown in blue signify thickening in HYPO (Control < HYPO).

It should be noted that as none of the analyses survived with smoothed data, results shown presently are based on unsmoothed findings. Table 2 provides results from the between-group comparisons, while Figure 1 shows spatial maps of the regions differentiating groups. Because HYPO was compared against controls (i.e., Control minus HYPO), red clusters signify regions of significant *thinning* in HYPO (Controls > HYPO as per heat bars) while blue clusters signify regions of significant *thickening* (Controls < HYPO). HYPO demonstrated five regions that were significantly thinner than in controls, two in the left hemisphere (viz., insula and fusiform gyrus) and three in the right (viz., frontomarginal sulcus, inferior parietal gyrus, and precuneus). By contrast, HYPO demonstrated eight regions (five left, three right) of significant thickening compared with controls (Figure 1, blue clusters). In the left hemisphere, thickening was primarily frontal (positive *y*-value in coordinates in Table 2) and/or superior (high positive *z*-value), whereas in the right, it was posterior (negative *y*-value) or inferior (negative *z*-value). As Table 2 indicates, right-hemisphere thickening was confined to temporal and occipital regions, namely the temporal pole, inferior temporal gyrus, and pericalcarine sulcus (i.e., occipital pole).

**Table 2:** **Cortical regions showing differences between HYPO (*n* = 20) and control (*n* = 24) groups^a^**.

Hemisphere	Region	Lobe	Brodmann area	Coordinates^b^	Size (mm^3^)	Number of vertices	*p*-value
**Thinning (HYPO < Control)^c^**							
Left	Insula	Frontal	13	−37, −15, 8	108.4	220	0.008
	Fusiform gyrus	Temporal	37	−32, −57, −11	93.8	146	0.026
Right	Frontomarginal sulcus	Frontal	10	25, 48, −4	90.8	137	0.029
	Inferior parietal gyrus	Parietal	39	53, −48, 28	97.6	210	0.015
	Precuneus gyrus	Parietal	31	15, −48, 35	86.8	265	0.04
**Thickening (HYPO > Control)^d^**							
Left	Rostral middle frontal gyrus	Frontal	10	−32, 46, 13	105.6	155	0.007
	Superior frontal gyrus	Frontal	6	−19, −4, 58	160.0	315	0.0002
	Superior frontal gyrus	Frontal	6	−10, −7, 56	109.8	244	0.005
	Superior parietal gyrus	Parietal	7	−11, −64, 53	88.9	178	0.028
	Postcentral sulcus	Parietal	2	−54, −18, 31	90.2	187	0.025
Right	Temporal pole	Temporal	38	41, 11, −32	138.9	203	0.0008
	Inferior temporal gyrus	Temporal	37	48, −36, −17	115.7	232	0.003
	Pericalcarine sulcus	Occipital	17	19, −69, 13	91.6	162	0.03

*^a^Excluded from the original HYPO group were two children with unacceptable scans and two whose mothers’ TSH values were in the normal range throughout pregnancy*.

*^b^Talairach coordinates (*x*, *y*, *z*) provide a three-dimensional atlas system that serves to map locations of specific brain structures; the *x*-coordinate indicates location in the right (positive)/left (negative) direction, *y* indicates anterior (positive)/posterior (negative) direction, and *z* signifies superior (positive)/interior (negative) direction*.

*^c^Shown as red tones in Figure 1 to denote Control > HYPO, as per heat bars*.

*^d^Shown as blue tones in Figure 1 to denote Control < HYPO*.

### Within-Group Analyses by Severity of Maternal Hypothyroidism

Two series of analyses were conducted, one examining differences between Mild and Severe subgroups and the other correlating trimester-specific maternal TSH levels with CT. In order to capture a broad range of TSH values and increase sample size, we included all HYPO cases but excluded the child whose mother lacked actual TSH data and only reported having been severely hypothyroid for a final sample size of 21 (10 Mild and 11 Severe).

**Figure 2:**
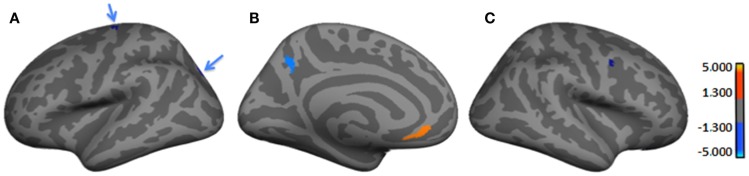
**Regions showing significant cortical thickness differences between Severe and Mild HYPO subgroups**. **(A)** Left lateral-view spatial map showing two regions (blue arrows) of significant thickening (blue) in Severe subgroup. **(B)** Left medial-view spatial map showing regions of significant thickening (blue) and thinning (orange/red) in Severe subgroup; **(C)** Right lateral-view spatial map showing a region of significant thickening (blue) in Severe subgroup. As per heat bars, regions depicted in red signify thinning in the Severe subgroup (i.e., Mild > Severe) and regions shown in blue signify thickening (i.e., Mild < Severe). Note: significant subgroup differences in left fusiform gyrus, right orbital gyrus, and right lateral orbital sulcus were not evident in current views.

Table 3 and Figure 2 provide the subgroup-comparison results. The Severe subgroup showed *thinning* in two left- and one right-hemisphere regions relative to Mild (Figure 2 red clusters as per heat bar) and *thickening* in three left- and two right-hemisphere regions (Figure 2 blue clusters). Thinning in the Severe subgroup was largely frontal or inferior (e.g., right orbital gyrus, left fusiform gyrus), whereas their *thickening* differed by hemisphere. Specifically, the Severe subgroup showed more extensive thickening than Mild in the left hemisphere spanning from frontal to occipital lobes. Right-hemisphere thickening was confined to frontal regions.

**Table 3:** **Regions showing cortical thickness differences between Severe (*n* = 11) and Mild (*n* = 10)^a^ HYPO subgroups^b^**.

Hemisphere	Region	Lobe	Coordinates^c^	Size (mm^2^)	Number of vertices	*p*-value
**Thinning (Severe < Mild)^d^**						
Left	Medial orbitofrontal sulcus	Frontal	−7, 25, −11	108.88	223	0.0002
	Fusiform gyrus	Temporal	−40, −70, −11	53.80	80	0.03
Right	Orbital gyrus	Frontal	24, 10, −15	54.22	145	0.03
**Thickening (Severe > Mild)^e^**						
Left	Precentral gyrus	Frontal	−21, −15, 65	56.06	150	0.025
	Precuneus gyrus	Parietal	−8, −54, 38	118.68	213	0.0002
	Superior occipital sulcus	Occipital	−22, −74, 34	74.79	110	0.002
Right	Precentral gyrus	Frontal	44, 2, 37	51.08	81	0.05
	Lateral orbital sulcus	Frontal	19, 22, −17	54.47	100	0.03

*^a^Sample size includes the two children whose mothers had normal-range TSH values throughout pregnancy but excludes the one child whose mothers’ specific TH value information was missing*.

*^b^Defined as TSH above or below 8.8 mU/l in first or second trimesters. Ranges: Mild = 0.1–8.4; Severe = 8.8–38.5 mU/l*.

^c^See description of Talairach coordinates in Table 2.

*^d^Shown as red tones in Figure 2 to denote Mild > Severe*.

*^e^Shown as blue tones in Figure 2 to denote Mild < Severe*.

**Figure 3:**
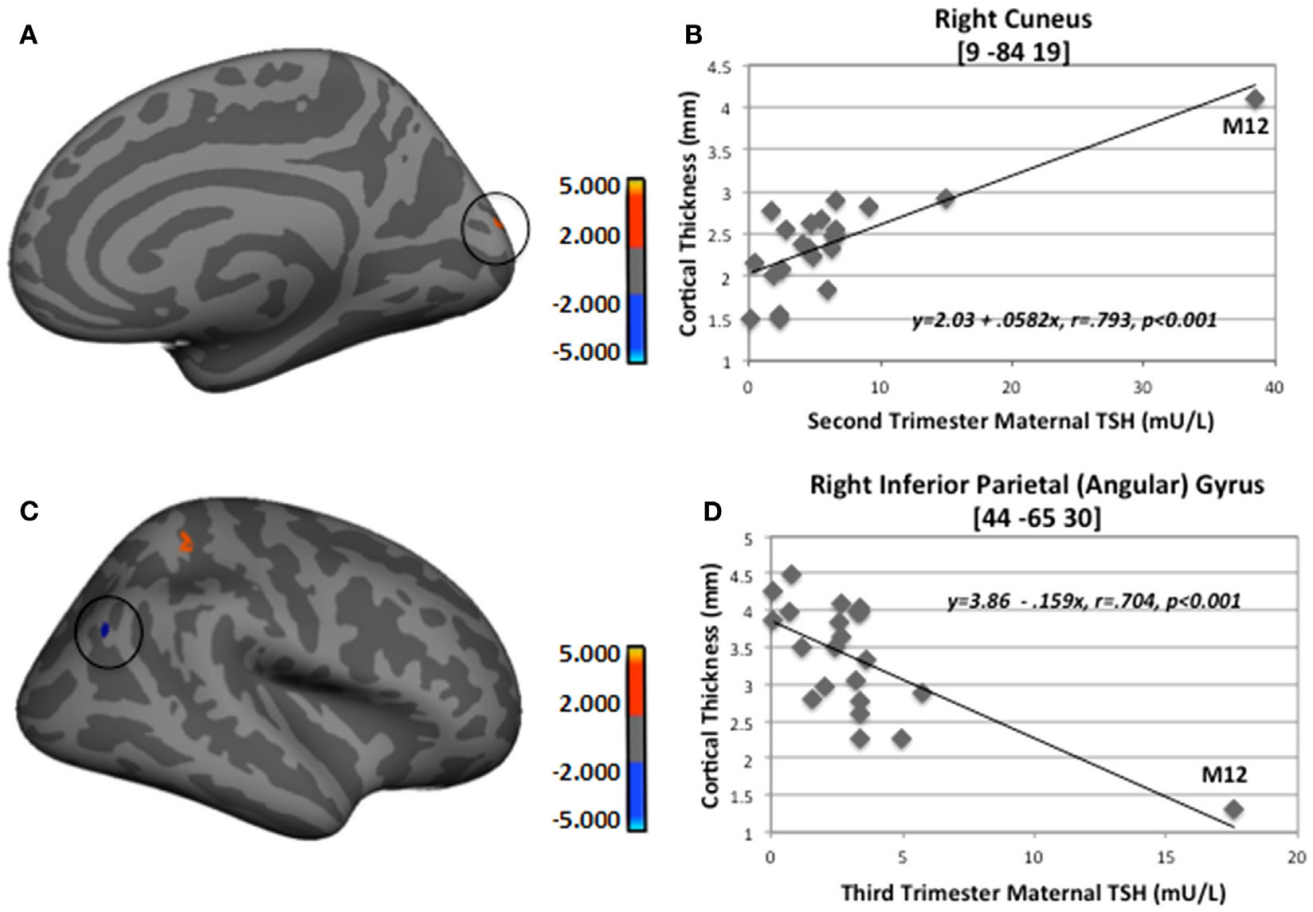
**Sample regions showing significant correlations between CT and second- and third-trimester maternal TSH**. **(A)** Right medial-view spatial map showing increased thickening (red) in right cuneus as maternal second-trimester TSH increased. **(B)** Correlation plot between individual CT values and second-trimester maternal TSH levels in right cuneus. **(C)** Right lateral-view spatial map showing increased thinning (blue) in right inferior parietal angular gyrus (circled) as third-trimester maternal TSH increased. **(D)** Correlation plot between individual CT values and third-trimester maternal TSH levels in right inferior parietal angular gyrus. Note, these analyses included M12 outlier; however, when M12 was not included in FreeSurfer analyses, thinning in right inferior parietal angular gyrus still remained (*p* = 0.01).

To assess the impact of degree of maternal hypothyroidism severity on cortical morphology, we conducted regression analyses in FreeSurfer between maternal TSH levels and CT. Regressions were performed on the 23 HYPO with TSH data. In addition, to check if the child whose mother consistently had very elevated TSH levels was driving any of the regression effects, we repeated these analyses excluding M12. Table 4 presents the regression results for the full HYPO group, while Table 5 contains the results without M12. In Figure 3 are shown sample findings of instances of thickening (positive correlations represented as red clusters in spatial maps) and thinning (negative correlations represented as blue clusters).

**Table 4:** **Regions showing significant correlations (*p* < 0.01) between maternal TSH values by trimester and vertex-based cortical thickness results for entire HYPO group**.

Region	Lobe	Effect	Coordinates	Number of vertices	Cluster-wise *p*-value
**Trimester 1**					
***L Precentral sulcus***	***Frontal***	***Thickening***	***−15, −13, 60***	***91***	***0.004***
R Supramarginal gyrus	Parietal	Thickening	46, −26, 24	93	0.005
R Supramarginal gyrus	Parietal	Thickening	53, −39, 35	68	0.006
***R Cuneus***	***Occipital***	***Thickening***	***7, −84, 20***	***43***	***0.005***
**Trimester 2**					
***L Precentral gyrus***	***Frontal***	***Thickening***	***−33, −16, 62***	***111***	***0.0002***
*R Postcentral sulcus*	*Parietal*	*Thickening*	*28, −39, 51*	*131*	*0.0002*
***R Cuneus***	***Occipital***	***Thickening***	***8.5, −84, 19***	***64***	***0.0002***
**Trimester 3**					
L Pericallosal sulcus	Temporal	Thinning	−6, 11, 24	99	0.008
R Inferior angular gyrus	Parietal	Thinning	44, −65, 30	58	0.007
***L Precentral sulcus***	***Frontal***	***Thickening***	***−15, 12, 59***	***91***	***0.003***
*R Postcentral sulcus*	*Parietal*	*Thickening*	*28, −39, 51.5*	*135*	*0.002*
L Inferior occipital gyrus/sulcus	Occipital	Thickening	−31, −86, −1	42	0.008
***R Cuneus***	***Occipital***	***Thickening***	***12, −86, 18***	***53***	***0.008***

*Values in bold italics indicate relationship maintained through three trimesters and in italics only, relationship maintained in two consecutive trimesters*.

**Table 5:** **Regions showing significant correlations (*p* < 0.01) between maternal TSH values and vertex-based cortical thickness results with primary outlier removed**.

Region	Lobe	Effect	Coordinates	Number of vertices	Cluster-wise *p*-value
**Trimester 1**					
*R Inferior supramarginal gyrus*	*Parietal*	*Thickening*	*53, *−*40, 35*	*76*	*0.0018*
**Trimester 2**					
R Middle temporal gyrus	Temporal	Thinning	60, −49, 1	90	0.0002
L Inferior angular gyrus	Parietal	Thinning	−45, −68, 27	58	0.004
L Precuneus gyrus	Parietal	Thinning	−8, −69, 43	67	0.007
L Inferior frontal gyrus	Frontal	Thickening	−54, 20, 14	63	0.009
*L Pericallosal sulcus*	*Temporal*	*Thickening*	*−36, −27, 9*	*92*	*0.006*
L Postcentral sulcus	Parietal	Thickening	−33, −33, 52	78	0.003
**Trimester 3**					
L Insula circular sulcus	Frontal	Thickening	−27, 18, −16	731	0.008

*Values shown in italics are also seen in Table 4, signifying effect remains with outlier removed*.

For the first set of regressions with M12 included, Table 4 indicates mainly positive relationships signifying more thickening than thinning as maternal TSH levels increased. First- and second-trimester TSH levels were only associated with thickening, whereas third-trimester maternal TSH levels were associated with both thinning and thickening. The thinning associated with third-trimester TSH was seen in the, left pericallosal sulcus and right inferior angular gyrus. Most regions that became thicker as maternal TSH levels increased were located frontally in the left hemisphere and more posteriorly in the right. Two of the regions showing thickening, namely left precentral sulcus/gyrus and right cuneus, showed significant correlations with TSH levels from all three trimesters while one region, right postcentral sulcus, showed positive correlations with second- and third-trimester TSH levels. Figure 3 provides sample findings for both effects. The upper panel shows thickening in the right cuneus as maternal second-trimester TSH levels increased while the lower panel shows thinning of the right inferior parietal angular gyrus as maternal third-trimester TSH levels increased.

**Figure 4:**
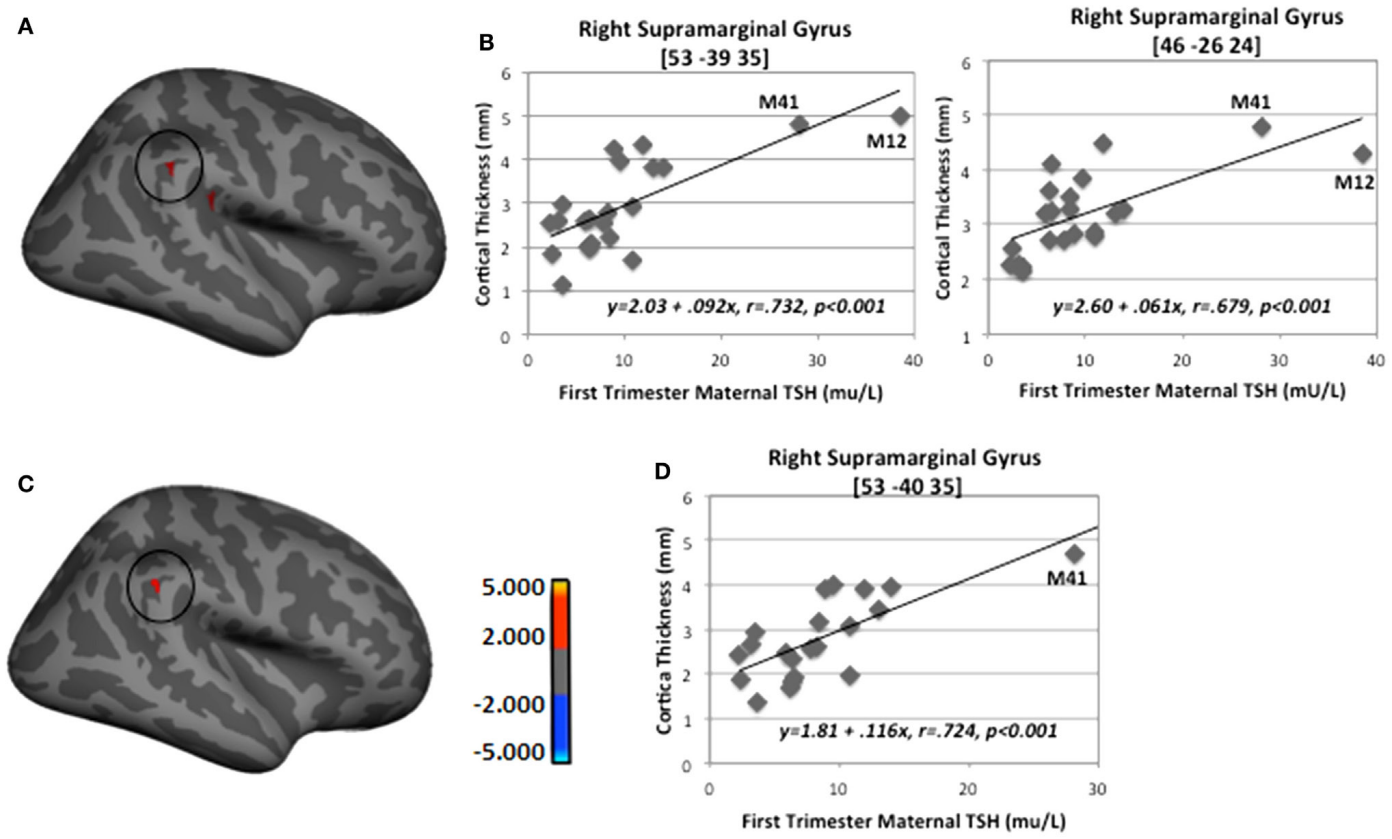
**Sample regions showing significant correlations between CT and first-trimester maternal TSH**. **(A)** Right lateral-view spatial map showing two nearby regions, indicating significant (*p* < 0.001) thickening (red) as first-trimester maternal TSH levels increased. **(B)** Correlation plots between individual CT values and first-trimester maternal TSH levels in two regions of right supramarginal gyrus. **(C)** Right lateral-view spatial map showing significant thickening (red) as first-trimester maternal TSH levels increased with M12 removed from FreeSurfer analysis. **(D)** Correlation plot between individual CT values and first-trimester maternal TSH levels in right supramarginal gyrus with M12 removed from FreeSurfer analysis. Note: correlations were retained when both outlying cases (M12 and M41) were removed from SPSS analyses shown in B (*p* = 0.006 and *p* = 0.01) and when M41 was removed from analysis shown in D (*p* = 0.002).

When M12’s data were removed from the regression analysis, only two of the regions remained, namely right inferior supramarginal gyrus with first-trimester TSH levels and left pericallosal sulcus with second-trimester (see Table 5). Figure 4 depicts the findings between first-trimester maternal TSH and right supramarginal gyrus CT including (upper panel) and excluding (lower panel) the data from M12. The similar patterns across both sets of figures indicate the effect was not driven by this outlier, signifying that even mildly elevated maternal TSH can disrupt cortical development in this region.

A supplementary series of simple correlations was conducted between trimester-specific maternal TSH values and individually extracted CT values at the peak vertex for each of the significant clusters listed in Table 4; values were derived from the FreeSurfer analysis conducted with all HYPO participants included. Table 6 shows results for three series of correlations performed in SPSS (version 23), one using the entire HYPO group, one eliminating M12 from the analyses, and a third eliminating both M12 and another less severe outlier (M41). Notably, many but not all correlations remained when the outliers were removed, particularly those involving analyses with first- and second-trimester TSH levels.

**Table 6:** **Coefficients for correlation analyses between maternal TSH and individually extracted CT values performed with and without outliers**.

Region	Lobe	Coordinates	Total Sample	Without M12	Without M12 and M41
**First-trimester tsh**					
*Thickening*					
L Precentral sulcus	Frontal	−15, −13, 60	0.725***	0.468*	0.255
R Supramarginal gyrus	Parietal	45, −26, 24	0.679***	0.699***	0.553**
R Supramarginal gyrus	Parietal	53, −39, 35	0.732***	0.687***	0.595**
R Cuneus	Occipital	7, −84, 20	0.708**	0.737**	0.561*
**Second-trimester tsh**					
*Thickening*					
L Precentral gyrus	Frontal	−33, −16, 62	0.764***	0.453*	0.462*
R Postcentral sulcus	Parietal	28, −39, 57	0.972***	0.403	0.406
R Cuneus	Occipital	8.5, −84, 19	0.793***	0.611**	0.614**
**Third-trimester tsh**					
*Thinning*					
L Pericallosal sulcus	Temporal	−6, 11, 24	−0.700***	−0.366	−0.395
R Inferior angular gyrus	Parietal	44, −65, 30	−0.704***	−0.548**	−0.496*
*Thickening*					
L Precentral sulcus	Frontal	−15, 12, 59	0.738***	0.405	0.469*
R Postcentral sulcus	Parietal	28, −39, 52	0.815***	0.074	0.045
L Inferior occipital gyrus/sulcus	Occipital	−31, −86, −1	0.557**	0.175	0.119
R Cuneus	Occipital	12, −86, 18	0.745***	0.273	0.253

All results obtained from FreeSurfer analyses performed on entire sample, as shown in Table 4; ***p < 0.001; **p < 0.01; *p < 0.05.

## Discussion

Current findings provide preliminary support for our hypothesis that children exposed gestationally to insufficient TH due to mother’s hypothyroidism will show atypical cortical development reflecting the severity and timing of her condition. We found that when they were compared with children born to normothyroid women, the offspring of women with HYPO showed a number of cortical regions that were thinner or thicker than in controls. Thinning was bilateral and in frontal, parietal, and temporal regions, whereas thickening differed by hemisphere and tended to be superior in the left hemisphere and inferior or posterior (e.g., temporal and occipital poles) in the right. However, only one of the regions differentiating groups was associated with severity of hypothyroidism in the mother (based on subgroup analysis), namely left fusiform gyrus. Correlations between mothers’ trimester-specific TSH levels and CT values revealed a greater number of positive than negative effects, signifying greater thickening from more severe maternal hypothyroidism. While some effects were driven by one or two outliers born to women with the most extreme HYPO, a number of other effects remained when these children were removed from the analyses, suggesting that even a mild degree of maternal hypothyroidism can disturb offspring cortical development.

Cortical thinning in HYPO was observed in left fusiform gyrus and insula and in right frontomarginal sulcus, inferior parietal gyrus, and precuneus. Stratification by severity of maternal hypothyroidism (in first half of pregnancy) revealed that, relative to the Mild subgroup, the Severe subgroup manifested thinning in left fusiform gyrus and also left orbitofrontal sulcus and right orbital gyrus. Consistent findings from between- and within-group analyses in the left fusiform gyrus suggest that this region is quite sensitive to severe maternal hypothyroidism, as the Severe subgroup was likely driving the difference with controls. However, the other regions differentiating HYPO and control groups (e.g., left insula) not seen in subgroup comparisons may mean either no effect of hypothyroidism severity (based on early-pregnancy maternal TSH levels) or our sample size was too small for the subgroup analysis to show significant differences. By contrast, the Severe subgroup showed thinning relative to Mild in inferior frontal areas (viz., left medial orbitofrontal sulcus and right orbital gyrus), signifying that since stratification was based on mothers’ TSH levels from the first half of pregnancy, some anterior parts of the cortex had a very early need for TH. The thinning observed across analyses suggests that both early and later aspects of corticogenesis may be disrupted by maternal hypothyroidism (32).

Explanations for thinning based on rodent evidence suggest that several key developmental processes may be disturbed by TH insufficiency from hypothyroidism early in gestation. For example, symmetric division may have failed to stop leading to an accumulation of neurons in the ventricular zone with fewer ultimately reaching the cortical mantle (25). Alternatively, some early effects may reflect neuronal migration abnormalities due to increased tangential and less radial migration (21, 22) with the result that fewer neurons ultimately reach certain cortical regions and others have more neurons (see below). Also, disturbances in the antioxidant defense system can lead to reduced survival of proliferating neurons during early gestation, as also seen in a murine model of maternal hypothyroidism (55). In rodents, maternal TH insufficiency later in gestation also affects more advanced cortical processes, such as dendritic arborization, which is reduced (21), and apoptosis, which is increased (25). Overall, these findings suggest that, in humans, multiple developmental processes are likely perturbed by maternal hypothyroidism, while specific effects reflect the exact timing of mother’s TH deficiency in relation to ongoing non-uniform brain development. Accordingly, all of these effects can give the appearance in FreeSurfer of a thinner cortex in specific regions.

Children born to hypothyroid mothers also showed extensive thickening across the entire cortical mantle; thickening was more prevalent than thinning. Within the left hemisphere, effects were predominantly in superior and anterior regions and in the right hemisphere, within inferior and posterior regions (Table 2). Within-group analyses revealed that the Severe subgroup had thickening across the left cortex relative to Mild but this occurred only frontally in the right hemisphere (Table 3). By contrast, regression results showed that maternal TSH levels were primarily correlated with CT in left frontal regions, whereas effects were more widespread in the right hemisphere. Although there is no ready explanation for these contradictory findings between the two sets of analyses, these findings may suggest inter-hemispheric differences in timing of need for TH. Since effects were primarily posterior in the right hemisphere and since developmental progression seems to be in a posterior-to-anterior direction, this suggests the right hemisphere may lag behind the left both developmentally and in vulnerability to hypothyroidism. Separate regressions with and without outliers also suggest different specific effects depending on whether hypothyroidism is severe or not. With outliers removed, the observed thickening in regions such as the right supramarginal gyrus and precentral gyrus suggests that mild elevations in TSH among treated hypothyroid women can have a significant and sustained impact on the developing fetal brain.

Explanations for cortical thickening based on rodent evidence may also reflect different aspects of cortical migration. First, due to diminishment of the glial scaffold needed for radial migration toward the cortical plate (22), some neurons may have remained in lower layers or even WM, thus giving the appearance of a longer – and hence thicker – human cortex. Alternatively, the increased tangential and decreased radial migration associated with TH insufficiency may mean that some regions had augmented number of neurons as opposed to others that had fewer neurons (see above discussion on thinning and migration). Second, it is also possible that the observed cortical thickening reflected alterations in the boundaries between GM and WM, given that unmyelinated peripheral axonal fibers look similar to GM (56). Finally, since maternal hypothyroidism is known to disrupt the balance between astrocytes and oligodendrocytes (i.e., fewer oligodendrocytes, more astrocytes) (57), this may mean that the number of exposed unmyelinated axons is increased leading to more GM than WM, which in FreeSurfer can also appear as increased thickening of the cortex.

Lateralized differences in thinning and thickening may reflect timing of need for TH given the unique cell compositions and different rates of development of the right and left hemispheres (58, 59). These laterality effects are to our knowledge unique and not similarly reported in the rodent literature. Additionally, since different cortical regions vary as to phases of cell development and positioning when cell cycle is completed and apoptosis occurs (25), this may explain our unexpected findings also of both thinning and thickening in nearby regions (e.g., right orbital gyrus and right lateral orbital gyrus, see Table 3).

It is relevant to note we recently reported that children with CH, who underwent a brief circumscribed period of TH insufficiency that occurred somewhat later and extended longer than in HYPO, also showed that cortical thinning and thickening relative to controls and effects reflected the severity of their hypothyroidism (45). Both CH and HYPO conditions showed thinning of the right precuneus and thickening in right occipital regions, while left precentral gyrus and right cuneus and postcentral gyrus thickening were correlated with late gestational or perinatal TH elevations in both populations. However, CH showed more instances of thinning than thickening compared with HYPO, particularly in the frontal lobes (as well as temporal pole and inferior temporal gyrus) and on regression analyses showed more negative correlations with TSH levels. Differences between CH and HYPO studies may reflect their different timing of TH insufficiency, which was later in CH. Thus, the increased thinning in CH versus HYPO may reflect the impact of their later TH loss on more protracted aspects of cortical formation, such as process growth and apoptosis. On the other hand, differences between studies may reflect the slightly older ages of the CH group and their matched controls than the HYPO group and their controls, especially given longitudinal evidence that thickening normally occurs up to a specific age in puberty followed by thinning (60).

Our observations of a broad range of cortical regions with atypical morphology in the offspring of hypothyroid women have important implications for the daily functioning of their children. For example, their thinning in the right fusiform gyrus may contribute to face- and object-processing difficulties, while effects in the right precuneus may be associated with weak spatial and math skills (4, 47, 61). Also, their cortical thinning in right precuneus and frontomarginal sulcus can contribute to the autobiographical memory weaknesses we previously noted in this population (62, 63). On the other hand, increased thickening in posterior structures may correspond to our previous findings that these children had visual processing difficulties, including poor contrast sensitivity, when mothers were severely hypothyroid (64); this may also lead to the observed non-verbal difficulties following maternal hypothyroxinemia (46). Observed thickening in frontal regions may contribute to problems in executive functioning, including difficulties in sustaining and maintaining attention (4, 8, 65) and increased risk of attention-deficit disorder and autism (10, 65). Finally, our finding of thickening within the right supramarginal gyrus of the parietal lobe has important implications for social functioning, given this region is critical for empathy and emotion processing (66). Furthermore, this finding may be a prelude to recent reports of increased emotional problems they exhibit as adults (67, 68). Interestingly in a preliminary analysis, we recently observed a positive correlation between degree of thickening and their emotion regulation in our adolescent sample. Our findings also revealed that thickening effects in some regions, such as the postcentral gyrus and sulcus and pericallosal sulcus, which are important for sensory processing, but these disappeared once outliers were removed, suggesting that central regions may be especially vulnerable to severe TH deficiency in pregnancy.

Present results also have important clinical significance for pregnant women whose offspring may be affected by mild elevations in maternal TSH depending on trimester of TH deficiency. Consequently, it is important to closely and frequently monitor women with hypothyroidism in order to ensure their dosages are sufficient to maintain their TH levels within the normal range throughout pregnancy. It is important to note that Korevaar and colleagues recently showed that for normothyroid women, those with early-to-mid pregnancy levels at the extremities of the normal range had children with lower IQs and smaller cortical volumes (47).

Although this study is unique because it includes offspring of hypothyroid women followed for a minimum of 10–12 years from or shortly after pregnancy and detailed assessments (described elsewhere) and structural (14, 15) and functional (in progress) MRI scans, our study is also subject to a number of limitations. Our small sample size represents an approximately two-thirds loss from the original group; however, no bias was observed in those who dropped out versus those who participated currently. Nevertheless, this has meant we could not analyze for other factors, such as sex or age, which can influence CT (69, 70). Also, the age range of our sample spans when the normal age-trajectory for cortical thickening reaches its peak and then starts to thin (60, 71); consequently, HYPO’s observed thickening can reflect either a permanent defect or a delay in development relative to controls. Likewise, we did not assess for pubertal maturity, which additionally affects CT (69). Furthermore, although we had thyroid information on most mothers, many had only TSH data and in most cases, only a single measurement per trimester. Consequently, we could not examine for degree of variability within a trimester and as we used averaged values within each trimester (when a mother provided more than one measurement per trimester), we could not examine for the impact of very low or very high TH levels. We also could not examine for effects of hypothyroxinemia. While our control group came from ostensibly normothyroid women who were ascertained similarly to the HYPO group, we lacked direct evidence they were in fact euthyroid, except for a small group of normothyroid women who were assessed and showed normal levels. Finally, as this study was preliminary, results were not correlated with indices of neuropsychological functioning and this needs to be done in future research on this population.

Several methodological issues may also influence our findings. Scans were obtained on a 1.5-T scanner, for which resolution between cortical layers may be less clear than with higher Tesla scanners, while analyses were performed using an early version of FreeSurfer, which may yield CT differences from later versions. Also, FreeSurfer (34) uses an adult template to label gyri and sulci and as our participants were pre-adolescents, some regional differences may not accurately reflect those designated by the coordinates in the output. In addition, as our results are based on unsmoothed and hence noisy data, some results may be false positives. Furthermore, we performed correlations between maternal TSH and CT measurements derived from the peak vertex only, not CT averaged across the identified cluster, and this may give an incomplete picture of the full impact of maternal hypothyroidism on offspring cortical development.

## Conclusion

Young adolescents exposed to insufficient maternal TH *in utero* show abnormal cortical development reflecting both thinning and thickening in different regions. Thickening was more common and extensive than thinning and appeared to reflect early TH insufficiency. Since specific regional and hemispheric differences depended on the trimester of TH insufficiency, this may reflect the spatiotemporal dynamics of the developing human brain in its need for TH during gestation. However, given the limitations and our preliminary approach, further research using larger samples and in relation to other gestational TH deficiencies (e.g., resistance to TH, hypothyroxinemia of prematurity) or forms of maternal TH insufficiency (e.g., subclinical hypothyroidism, hypothyroxinemia, iodine deficiency) is warranted. Future studies also need to examine the relations between aspects of cortical development and higher-order cognitive and sociobehavioral functions in order to better characterize the full impact of maternal hypothyroidism on the progeny. Nevertheless, our findings do signify that TH plays a central in human corticogenesis.

## Author Contributions

JR conceptualized, obtained funding, and managed the original study from which scans were derived. JEL, HC, and JR conceived the current study. JS conducted the FreeSurfer analyses. JEL, JS, and interpreted the data. JEL under the supervision of JR drafted the first manuscript version, while JS and HC conducted revisions to this draft. JR wrote the final manuscript version, while JEL, JS, and HC revised and approved this for submission for publication.

## Conflict of Interest Statement

The authors declare that the research was conducted in the absence of any commercial or financial relationships that could be construed as a potential conflict of interest.

## References

[1] Berbel P Navarro D Roman GC. An evo-devo approach to thyroid hormones in cerebral and cerebellar cortical development: etiological implications for autism. Front Endocrinol (2014) 5:146. 10.3389/fendo.2014.00146 25250016 PMC4158880

[2] Rovet JF. The role of thyroid hormones for brain development and cognitive function. Endocr Dev (2014) 26:26–43. 10.1159/000363153 25231442

[3] Rovet J Willoughby KA Maternal thyroid function and fetal brain development. In: Zimmerman A Connors S, editors. Maternal Influences on Fetal Neurodevelopment: Clinical and Research Aspects. New Jersey, NJ: Humana Press (2010). p. 55–77.

[4] Haddow JE Palomaki GE Allan WC Williams JR Knight GJ Gagnon J Maternal thyroid deficiency during pregnancy and subsequent neuropsychological development of the child. New Engl J Med (1999) 341:549–55. 10.1056/NEJM199908193410801 10451459

[5] Henrichs J Bongers-Schokking JJ Schenk JJ Ghassabian A Schmidt HG Visser TJ Maternal thyroid function during early pregnancy and cognitive functioning in early childhood: the Generation R Study. J Clin Endocrinol Metab (2010) 95:4227–34. 10.1210/jc.2010-0415 20534757

[6] Ghassabian A Bongers-Schokking JJ Henrichs J Jaddoe VW Visser TJ Visser WE Maternal thyroid function during pregnancy and behavioral problems in the offspring: the Generation R Study. Pediatr Res (2011) 69:454–9. 10.1203/PDR.0b013e3182125b0c 21471776

[7] Ghassabian A Henrichs J Tiemeier H Impact of mild thyroid hormone deficiency in pregnancy on cognitive function in children: lessons from the Generation R Study. Best Pract Res Clin Endocrinol Metab (2013) 28:221–32. 10.1016/j.beem.2013.04.008 24629863

[8] Modesto T Tiemeier H Peeters RP Jaddoe VW Hofman A Verhulst FC Maternal mild thyroid hormone insufficiency in early pregnancy and attention-deficit/hyperactivity disorder symptoms in children. JAMA Pediatr (2015) 169:838–45. 10.1001/jamapediatrics.2015.0498 26146876

[9] Kooistra L Crawford S van Baar AL Brouwers EP Pop VJ. Neonatal effects of maternal hypothyroxinemia during early pregnancy. Pediatrics (2006) 117:161–7. 10.1542/peds.2005-0227 16396874

[10] Román GC Ghassabian A Bongers-Schokking JJ Jaddoe VW Hofman A de Rijke YB Association of gestational maternal hypothyroxinemia and increased autism risk. Ann Neurol (2013) 74:733–42. 10.1002/ana.23976 23943579

[11] Berbel P Bernal J Hypothryoxinemia: a subclinical condition affecting neurodevelopment. Expert Rev Endocrinol Metab (2010) 5:563–75. 10.1586/eem.10.37 30780800

[12] Pop VJ Brouwers EP Vader HL Vulsma V van Baar AL de Vijlder JJ. Maternal hypothyroxinaemia during early pregnancy and subsequent child development: a 3-year follow-up study. Clin Endocrinol (Oxf) (2003) 59:281–8. 10.1046/j.1365-2265.2003.01822.x 12919150

[13] Brent GA The debate over thyroid-function screening in pregnancy. N Engl J Med (2012) 366:562–3. 10.1056/NEJMe1112591 22316450

[14] Samadi A Skocic J Rovet JF. Children born to women treated for hypothyroidism during pregnancy show abnormal corpus callosum development. Thyroid (2015) 25:494–502. 10.1089/thy.2014.0548 25780811

[15] Willoughby KA McAndrews MP Rovet JF. Effects of maternal hypothyroidism on offspring hippocampus and memory. Thyroid (2014) 24:576–84. 10.1089/thy.2013.0215 24015847

[16] Morreale de Escobar G Obregon MJ Escobar del Rey F. Is neuropsychological development related to maternal hypothyroidism or to maternal hypothyroxinemia? J Clin Endocrinol Metab (2000) 85(11):3975–8. 10.1210/jcem.85.11.6961 11095417

[17] Zoeller RT Rovet J Timing of thyroid hormone action in the developing brain: clinical observations and experimental findings. J Neuroendocrinol (2003) 16(10):809–18. 10.1111/j.1365-2826.2004.01243.x 15500540

[18] Berbel P Navarro D Ausó E Varea E Rodríguez AE Ballesta JJ Role of late maternal thyroid hormones in cerebral cortex development: an experimental model for human prematurity. Cereb Cortex (2010) 20:1462–75. 10.1093/cercor/bhp212 19812240 PMC2871377

[19] Stenzel D Huttner WB. Role of maternal thyroid hormones in the developing neocortex and during human evolution. Front Neuroanat (2013) 7:19. 10.3389/fnana.2013.00019 23882187 PMC3712268

[20] Ruiz-Marcos A Sanchez-Toscano F Escobar del Rey F Morreale de Escobar G. Severe hypothyroidism and the maturation of the rat cerebral cortex. Brain Res (1979) 162:315–29. 10.1016/0006-8993(79)90292-0 761091

[21] Auso E Lavado-Autric R Cuevas E Escobar del Rey F Morreale de Escobar G Berbel P. A moderate and transient deficiency of maternal thyroid function at the beginning of fetal neocorticogenesis alters neuronal migration. Endocrinology (2004) 145(9):4037–47. 10.1210/en.2004-0274 15087434

[22] Cuevas E Auso E Telefont M Morreale de Escobar G Sotelo C Berbel P. Transient maternal hypothyroxinemia at onset of corticogenesis alters tangential migration of medial ganglionic eminence-derived neurons. Eur J Neurosci (2005) 22:541–51. 10.1111/j.1460-9568.2005.04243.x 16101736

[23] Berbel PJ Escobar del Rey F Morreale de Escobar G Ruiz-Marcos A. Effect of hypothyroidism on the size of spines of pyramidal neurons of the cerebral cortex. Brain Res (1985) 337:217–23. 10.1016/0006-8993(85)90057-5 4027570

[24] Navarro D Alvarado MC Navarrete F Giner M Obregon MJ Manznares J Gestational and early postnatal hypothyroidism alters VGluT1 and VGAT bouton distribution in the neocortex and hippocampus, and behavior in rats. Front Endocrinol (2015) 9:9. 10.3389/fnana.2015.00009 25741243 PMC4330898

[25] Mohan V Sinha RA Pathak A Rastogi L Kumar P Pal A Maternal thyroid hormone deficiency affects the fetal neocorticogenesis by reducing the proliferating pool, rate of neurogenesis and indirect neurogenesis. Exp Neurol (2012) 237:477–88. 10.1016/j.expneurol.2012.07.019 22892247

[26] Lucio RA Garcia JV Ramon Cerezo J Pacheco P Innocenti GM Berbel P. The development of auditory callosal connections in normal and hypothyroid rats. Cereb Cortex (1997) 7:303–16. 10.1093/cercor/7.4.303 9177762

[27] Berbel P Auso E Garcia-Velasco JV Molina ML Camacho M. Role of thyroid hormones in the maturation and organisation of rat barrel cortex. Neuroscience (2001) 107:383–94. 10.1016/S0306-4522(01)00368-2 11718994

[28] Auso E Cases O Fouquet C Camacho M Garcia-Velasco JV Gaspar P Protracted expression of serotonin transporter and altered thalamocortical projections in the barrelfield of hypothyroid rats. Eur J Neurosci (2001) 14:1968–80. 10.1046/j.0953-816x.2001.01815.x 11860492

[29] Lavado-Autric R Auso E Garcia-Velasco JV del Carmen Arufe M Escobar del Rey F Berbel P Early maternal hypothyroxinemia alters histogenesis and cerebral cotex cytoarchitecture of the progeny. J Clin Invest (2003) 111(7):1073–82. 10.1172/JCI200316262 12671057 PMC152582

[30] Rovet J Neurospychological follow-up of early-treated congenital hypothyroidism following newborn screening. In: Morreale de Escobar G de Vijlder J Butz S Hostalek U, editors. The Thyroid and Brain. Stuttgart: Schattauer (2003). p. 242–58.

[31] Gould E Butcher LL. Developing cholinergic basal forebrain neurons are sensitive to thyroid hormone. J Neurosci (1989) 9(9):3347–58. 2795167 10.1523/JNEUROSCI.09-09-03347.1989 PMC6569663

[32] Rakic P. A small step for the cell, a giant leap for mankind: a hypothesis of neocortical expansion during evolution. Trends Neurosci (1995) 18(9):383–8. 10.1016/0166-2236(95)93934-P 7482803

[33] Panizzon MS Fennema-Notestine C Eyler LT Jernigan TL Prom-Wormley E Neale M Distinct genetic influences on cortical surface area and cortical thickness. Cereb Cortex (2009) 19:2728–35. 10.1093/cercor/bhp026 19299253 PMC2758684

[34] Fischl B van der Kouwe A Destrieux C Halgren E Ségonne F Salat DH Automatically parcellating the human cerebral cortex. Cereb Cortex (2004) 14:11–2. 10.1093/cercor/bhg087 14654453

[35] Mak-Fan KM Taylor MJ Roberts W Lerch JP. Measures of cortical grey matter structure and development in children with autism spectrum disorder. J Autism Dev Disord (2012) 42(3):419–27. 10.1007/s10803-011-1261-6 21556969

[36] Raznahan A Lenroot R Thurm A Gozzi M Hanley A Spence SJ Mapping cortical anatomy in preschool aged children with autism using surface-based morphometry. Neuroimage Clin (2012) 2:111–9. 10.1016/j.nicl.2012.10.005 24179764 PMC3777762

[37] Almeida LG Ricardo-Garcell J Prado H Barajas L Fernandez-Bouzas A Avila D Reduced right frontal cortical thickness in children, adolescents and adults with ADHD and its correlation to clinical variables: a cross-sectional study. J Psychiatr Res (2010) 44(16):1214–23. 10.1016/j.jpsychires.2010.04.026 20510424

[38] Raznahan A Cutter W Lalonde F Robertson D Daly E Conway GS Cortical anatomy in human X monosomy. Neuroimage (2010) 49(4):2915–23. 10.1016/j.neuroimage.2009.11.057 19948228 PMC3229914

[39] Fernandez-Jaen A Fernandez-Mayoralas DM Quinones Tapia D Calleja-Perez B Garcia-Segura JM Arribas SL Cortical thickness in fetal alcohol syndrome and attention deficit disorder. Pediatr Neurol (2011) 45(6):387–91. 10.1016/j.pediatrneurol.2011.09.004 22115001

[40] Yang Y Roussotte F Kan E Sulik KK Mattson SN Riley EP Abnormal cortical thickness alterations in fetal alcohol spectrum disorders and their relationships with facial dysmorphology. Cereb Cortex (2012) 22(5):1170–9. 10.1093/cercor/bhr193 21799209 PMC3328347

[41] Zhou D Lebel C Lepage C Rasmussen C Evans A Wyper K Developmental cortical thinning in fetal alcohol spectrum disorders. Neuroimage (2011) 58(1):16–25. 10.1016/j.neuroimage.2011.06.026 21704711

[42] Thompson PM Lee AD Dutton RA Geaga JA Hayashi KM Eckert MA Abnormal cortical complexity and thickness profiles mapped in Williams syndrome. J Neurosci (2005) 25(16):4146–58. 10.1523/JNEUROSCI.0165-05.2005 15843618 PMC6724948

[43] Lui JH Hansen DV Kriegstein AR. Development and evolution of the human neocortex. Cell (2011) 146(1):18–36. 10.1016/j.cell.2011.06.030 21729779 PMC3610574

[44] Rajaprakash M Chakravarty MM Lerch JP Rovet J Cortical morphology in children with alcohol-related neurodevelopmental disorder. Brain Behav (2014) 4:41–50. 10.1002/brb3.191 24653953 PMC3937705

[45] Clairman H Lischinsky J Skocic J Rovet J. Do children with congenital hypothyroidism exhibit abnormal cortical morphology? Pediatr Res (2015) 78:286–97. 10.1038/pr.2015.93 25978801

[46] Ghassabian A El Marroun H Peeters RP Jaddoe VW Hofman A Verhulst FC Downstream effects of maternal hypothyroxinemia in early pregnancy: nonverbal IQ and brain morphology in school-age children. J Clin Endocrinol Metab (2014) 99:2383–90. 10.1210/jc.2013-4281 24684462

[47] Korevaar TIM Muetzel R Medici M Chaker L Jaddoe VWV de Rijke YB Association of maternal thyroid function during early pregnancy with offspring IQ and brain morphology in childhood: a population-based prospective cohort study. Lancet Diabetes Endocrinol (2015) 4(1):35–43. 10.1016/S2213-8587(15)00327-7 26497402

[48] Alexander EK Marqusee E Lawrence J Jarolim P Fischer GA Larsen PR. Timing and magnitude of increases in levothyroxine requirements during pregnancy in women with hypothyroidism. New Engl J Med (2004) 351(3):241–9. 10.1056/NEJMoa040079 15254282

[49] Abalovich M Amino N Barbour LA Cobin RH De Groot LJ Glinoer D Management of thyroid dysfunction during pregnancy and postpartum: an Endocrine Society Clinical Practice Guideline. J Clin Endocrinol Metab (2007) 92(8 Suppl):S1–47. 10.1210/jc.2007-0141 17948378

[50] De Groot L Alexander EK Eastman C Rovet J Lazarus J Mestman J Endocrine society guideline on management of thyroid disease during pregnancy. J Clin Endocrinol Metab (2012) 97(8):2543–65. 10.1210/jc.2011-2803 22869843

[51] Azizi F Mehren L Amouzegar A Delshad H Tohidi M Askari S Establishment of the trimester-specific reference range for free thyroxine index. Thyroid (2013) 23:354–9. 10.1089/thy.2012.0407 23167270

[52] Wechsler D Wechsler Abbreviated Scale of Intelligence. New York: Psychological Corp (1999).

[53] Fischl B Dale AM. Measuring the thickness of the human cerebral cortex from magnetic resonance images. Proc Natl Acad Sci U S A (2000) 97(20):11050–5. 10.1073/pnas.200033797 10984517 PMC27146

[54] Dale AM Fischl B Sereno MI. Cortical surface-based analysis. I. Segmentation and surface reconstruction. Neuroimage (1999) 9(2):179–94. 10.1006/nimg.1998.0395 9931268

[55] Ahmed OM Ahmed RG El-Gareib AW El-Bakry AM Abd El-Tawab SM Effects of experimentally induced maternal hypothyroidism and hyperthyroidism on the development of rat offspring: II – the developmental pattern of neurons in relation to oxidative stress and antioxidant defense system. Int J Dev Neurosci (2012) 30:517–37. 10.1016/j.ijdevneu.2012.04.005 22664656

[56] Pathak A Sinha RA Mohan V Mitra K Godbole MM. Maternal thyroid hormone before the onset of fetal thyroid function regulates reelin and downstream signaling cascade affecting neocortical neuronal migration. Cereb Cortex (2011) 21(1):11–21. 10.1093/cercor/bhq052 20368265

[57] Sharlin DS Tighe D Gilbert ME Zoeller RT The balance between oligodendrobye and astrocyte production in major white matter tracts is linearly related to serum total thyroxine. Endocrinology (2008) 149:2527–36. 10.1210/en.2007-1431 18276755 PMC5393260

[58] Taylor DC Differential rates of cerebral maturation between sexes and between hemispheres. Lancet (1969) 2:140–2. 10.1016/S0140-6736(69)92445-3 4183249

[59] Chiron C Jambaque I Nabbout R Lounes R Syrota A Dulac O The right brain hemisphere is dominant in human infants. Brain (1997) 120:1057–65. 10.1093/brain/120.6.1057 9217688

[60] Gogtay N Giedd JN Lusk L Hayashi KM Greenstein D Vaituzis AC Dynamic mapping of human cortical development during childhood through early adulthood. Proc Natl Acad Sci U S A (2004) 101:8174–9. 10.1073/pnas.0402680101 15148381 PMC419576

[61] Noten AM Loomans EM Vrijkotte TG van de Ven PM van Trotsenburg AS Rotteveel J Maternal hypothyroxinaemia in early pregnancy and school performance in 5-year-old offspring. Eur J Endocrinol (2015) 173:563–71. 10.1530/EJE-15-0397 26306579

[62] Willoughby K Levine B McAndrews MP Effects of early thyroid hormone deficiency on children’s autobiographical memory performance. J Int Neuropsychol Soc (2013) 19(4):419–29. 10.1017/S1355617712001488 23369840

[63] Willoughby KA McAndrews MP Rovet JF. Accuracy of episodic autobiographical memory in children with early thyroid hormone deficiency using a staged event. Dev Cogn Neurosci (2014) 9:1–11. 10.1016/j.dcn.2013.12.005 24462783 PMC6989760

[64] Mirabella G Westall CA Asztalos E Perlman K Koren G Rovet J. Development of contrast sensitivity in infants with prenatal and neonatal thyroid hormone insufficiencies. Pediatr Res (2005) 57(6):902–7. 10.1203/01.PDR.0000157681.38042.B4 15774837

[65] Ghassabian A Bongers-Schokking JJ de Rijke YB van Mil N Jaddoe VW de Muinck Keizer-Schrama SM Maternal thyroid autoimmunity during pregnancy and the risk of attention deficit/hyperactivity problems in children: the Generation R Study. Thyroid (2012) 22:178–86. 10.1089/thy.2011.0318 22175242 PMC3271370

[66] Silani G Lamm C Ruff CC Singer T Right supramarginal gyrus is crucial to overcome emtoional egocentricity bias in social judgements. J Neurosci (2013) 33:15466–76. 10.1523/JNEUROSCI.1488-13.2013 24068815 PMC6618458

[67] Andersen SL Olsen J Wu CS Laurberg P. Psychiatric disease in late adolescence and young adulthood. Foetal programming by maternal hypothyroidism? Clin Endocrinol (Oxf) (2014) 91:126–33. 10.1111/cen.12415 24467638

[68] Gyllenberg D Sourander A Surcel J-M Hinkka-Yli-Somaki S McKeague IW Brown AS Hypothyroxinemia during gestation and offspring schizophrenia in a notional birth cohort. Biol Psychiatry (2015):1–9. 10.1016/j.biopsych.2015.06.014 PMC4684794 26194598

[69] Herting MM Gautam P Spielberg JM Dahl RE Sowell ER. A longitudinal study: changes in cortical thickness and surface area during pubertal maturation. PLoS One (2015) 10:e0119774. 10.1371/journal.pone.0119774 25793383 PMC4368209

[70] Sowell ER Peterson BS Kan E Woods RP Yoshii J Bansal R Sex differences in cortical thickness mapped in 176 healthy individuals between 7 and 87 years of age. Cereb Cortex (2007) 17:1550–60. 10.1093/cercor/bhl066 16945978 PMC2329809

[71] Shaw P Greenstein D Lerch J Clasen LS Lenroot R Gogtay N Intellectual ability and cortical development in children and adolescents. Nature (2006) 440:676–9. 10.1038/nature04513 16572172

